# Replication of hepatitis E virus in the ovary and promotion of oocyte apoptosis in rabbits infected with HEV-4

**DOI:** 10.18632/oncotarget.23381

**Published:** 2017-12-17

**Authors:** Junqing An, Tianlong Liu, Ruiping She, Qiaoxing Wu, Jijing Tian, Ruihan Shi, Wenzhuo Hao, Xinxin Ren, Yue Yang, Yiyao Lu, Yifei Yang, Yuanheng Wu

**Affiliations:** ^1^ Laboratory of Veterinary Pathology and Public Health, College of Veterinary Medicine, China Agricultural University, Beijing 100193, P.R. China

**Keywords:** hepatitis E virus, viral replication, apoptosis, ovary, vertical transmission

## Abstract

Hepatitis E virus (HEV) infection can induce infertility and miscarriage in pregnant women and infect neonates through vertical transmission. However, the mechanism of infertility and vertical transmission remains unclear. In the present study, we evaluated the replication of HEV in the ovary and structural and molecular changes induced by HEV after intraperitoneal injection of HEV in rabbits. Positive- and negative-strand HEV RNA was detected in the ovaries at 28 and 49 days post-infection. Positive HEV open reading frames 2 and 3 signals were observed in the ovaries by immunohistochemistry staining. Histopathological changes of ovarian tissues were observed, including scattered cell necrosis and lymphocyte infiltration. The ratio of normal follicles decreased, whereas the ratio of atresia follicles increased in the HEV RNA-positive ovaries compared to the control group by counting the number of follicles at all levels. In addition, TUNEL results showed that apoptosis in follicle cells and oocytes was promoted by HEV infection. These results suggest that the ovary is one of the replication sites of HEV and that the expression of HEV RNA and antigen in ovarian tissue caused structural and molecular changes that promoted germ cell apoptosis. HEV can infect and replicate in the ovum at different stages, which is a novel mechanism for HEV vertical transmission.

## INTRODUCTION

Hepatitis E (HE) caused by hepatitis E virus (HEV) is among the most important causes of epidemic and sporadic acute hepatitis worldwide [1]. HEV is a single-stranded positive-sense RNA virus that belongs to two genera of the hepeviridae family: orthohepevirus and pischihepevirus [2]. Four major genotypes of HEV that belong to the Orthohepevirus A species can infect humans (genotypes 1–4). Genotypes 1 and 2 only infect humans, whereas genotypes 3 and 4 are zoonotic [3]. The major transmission route of HEV is the fecal-oral route, which is mostly associated with contaminated water and uncooked meat [4]. In recent decades, extrahepatic lesions caused by HEV have attracted increasing attention because of their association with neurological symptoms, acute pancreatitis, severe thrombocytopenia, myositis, and kidney diseases [5–7]. Previous studies detected HEV RNA and antigens in the liver and extrahepatic tissues including the brain, salivary gland, small intestine, testis, and pancreas [8–10]. However, the mechanism of HEV infection and transmission remain unclear.

Recently, Xia and co-workers reported that HEV infection caused adverse outcomes in vertical transmission by experimentally infecting pregnant rabbits with a rabbit HEV isolate [11]. Although HEV is self-resolving and has a low fatality rate in the general population, the mortality rate in pregnant women is high [12]. Bose and co-workers demonstrated that HEV replicates in the placenta and its expression causes pathological injuries to the placenta [9]. HEV may undergo vertical transmission through the placenta. However, the role of the ovary in the vertical transmission of HEV is unknown. Because the ovary is the major organ of the female reproductive system, an understanding the mechanism of the ovary in miscarriage and infertility caused by HEV is urgently needed.

It is well-known that the disturbance of apoptosis is involved in the pathologic mechanisms of various diseases in humans. Some studies showed that the expression of HEV in the kidney and testis induces apoptosis and eventually leads to the pathological injury of these tissues [13, 14]. Numerous studies have shown that apoptosis is highly relevant to hepatitis caused by hepatitis B virus (HBV) and hepatitis C virus [15, 16]. However, whether expression of HEV in the ovary causes apoptosis of oocytes requires further investigation. Rabbits successfully infected with sHEV-4 were used in the present study [8]. We detected the HEV RNA, open reading frame (ORF)2, and ORF3 antigens and their distribution and localization in ovaries with the help of PCR and immunohistochemistry staining. Apoptotic cells were specifically stained using TdT-mediated dUTP nick-end labeling (TUNEL).

## RESULTS

### Positive- and negative-strand HEV RNA detection

In the control groups, all ovary samples were negative for HEV RNA. At 28 dpi, 1/4 ovary sample in experimental group was positive for the positive-strand HEV RNA detection. However, for the negative-strand HEV RNA detection, no ovary sample was positive. At 49 dpi, 2/4 ovary samples in the experimental groups were positive for both positive-strand HEV RNA and negative-strand detection. The results of positive ovary number for positive- and negative-strand HEV RNA detection at different time points are listed in Table 1. Images of the PCR results are listed in the supplementary materials (Supplementary Figures 1–3).

**Table 1:** The number of positive rabbits’ ovaries for positive- and negative-strand HEV RNA detection at different time points

Days post-inoculation (DPI)	Groups	The number of positive rabbits’ ovaries for positive- and negative-strand HEV RNA detection	
		Positive-strand HEV RNA detection	Negative-strand HEV RNA detection
28 dpi	Experimental groups	1/4	0/4
	Control groups	0/4	0/4
49 dpi	Experimental groups	2/4	2/4
	Control groups	0/4	0/4

### Real-time PCR detection of HEV RNA in ovaries

A standard curve was established as previously described by Yang [17]. The linear correlation (R^2^) between the CT and copy number logarithm was 0.985, slope of the standard curve was −3.201, and intercept was 47.665. The viral load of the ovary samples is summarized in Table 2. At 28 dpi, the viral load of the positive ovary was 1.10 × 10^3^ copies/mL. At 49 dpi, 2 ovary samples were positive and the viral loads were 2.06 × 10^4^ and 2.52 × 10^4^ copies/mL, respectively.

**Table 2:** Viral load of HEV RNA in rabbits’ ovaries at different time point

Days post-inoculation (DPI)	Groups	Viral load of HEV RNA in rabbits’ ovaries (Copies/mL)	
28 dpi	Experimental group	T1	N/A
		T2	1.10 × 10^3^
		T3	N/A
		T4	N/A
	Control group	C1	N/A
		C2	N/A
		C3	N/A
		C4	N/A
48 dpi	Experimental group	T5	2.06 × 10^4^
		T6	2.52 × 10^4^
		T7	N/A
		T8	N/A
	Control group	C5	N/A
		C6	N/A
		C7	N/A
		C8	N/A

N/A: negative for HEV RNA in ovaries.

### Expression of HEV antigen

**Figure 1:**
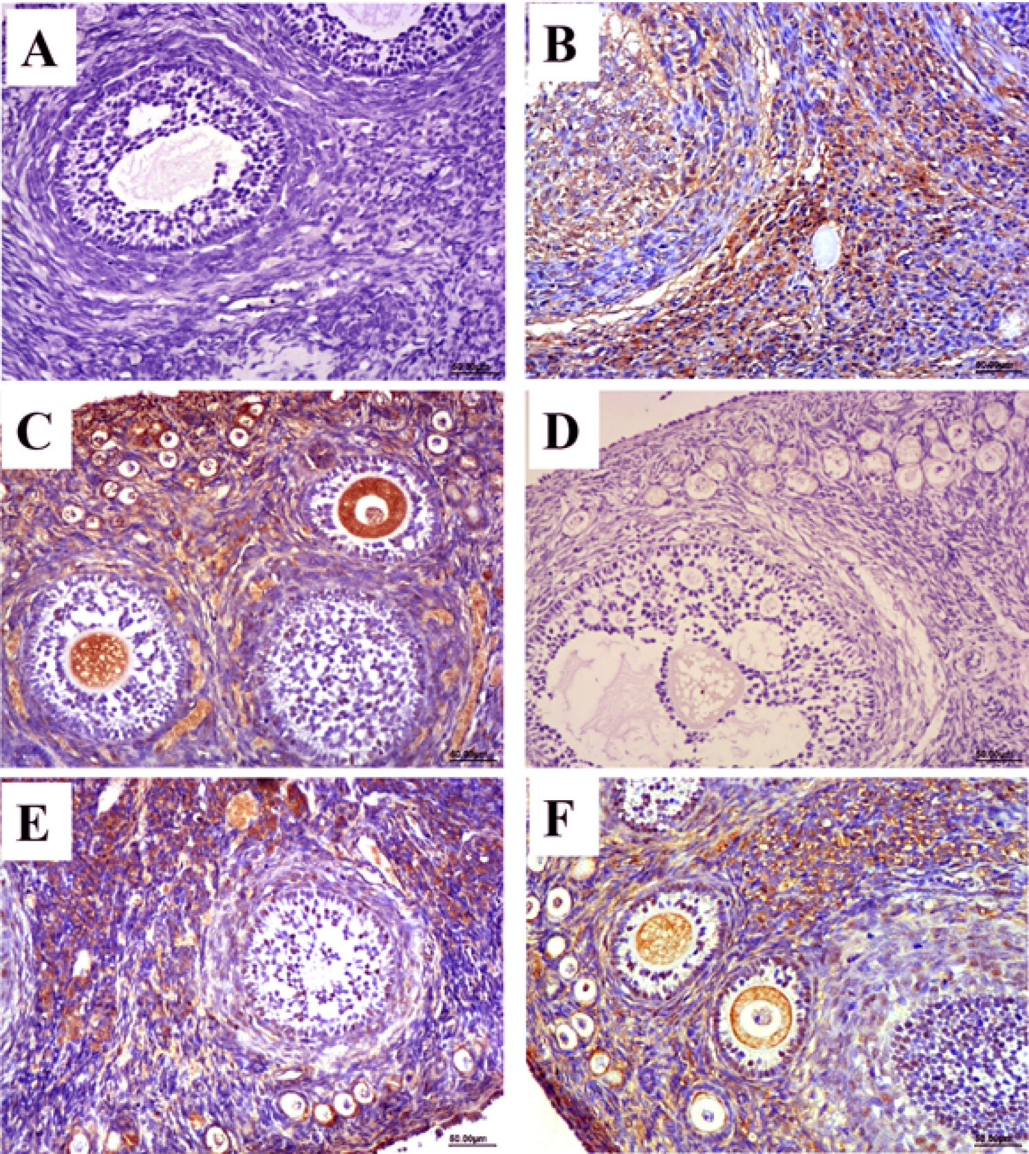
Immunohistochemical stain of HEV ORF2 and ORF3 antigen (**A**–**C**) Expression of HEV ORF2 in ovarian tissues, no positive signals in ovaries from control group (A). The positive signals distribute in the sperm cell and ovum cytoplasm of HEV-positive ovarian section at 28 dpi (B) and 49 dpi (C). (**D**–**E**) Expression of HEV ORF3 in ovarian tissues, no positive signals in ovaries from control group (D). The positive signals distribute in the sperm cell and ovum cytoplasm of HEV-positive ovarian section at 28 dpi (E) and 49 dpi (**F**). (Magnification: 20×).

The distribution and expression of HEV ORF2 and ORF3 in ovary tissues was observed by immunohistochemical staining. According to the immunohistochemical results, no positive signals for the HEV ORF2 and ORF3 antigens were detected in the ovary tissues in the control group (Figure 1A and 1D). In contrast, positive signals for ORF2 and ORF3 were observed in HEV RNA-positive ovaries in the experimental group at 28 and 49 dpi (Figure 1B–1F). The distribution of positive signals for HEV ORF2 and ORF3 in ovary tissues was similar. Positive signals were observed in the cytoplasm of interstitial cells of the ovary, follicular cells of primordial and primary follicles, and granulosa cells of secondary follicles. Importantly, we also found positive signals in the ovarian ovum (Figure 1B–1F).

### Histopathological observation of ovary

**Figure 2:**
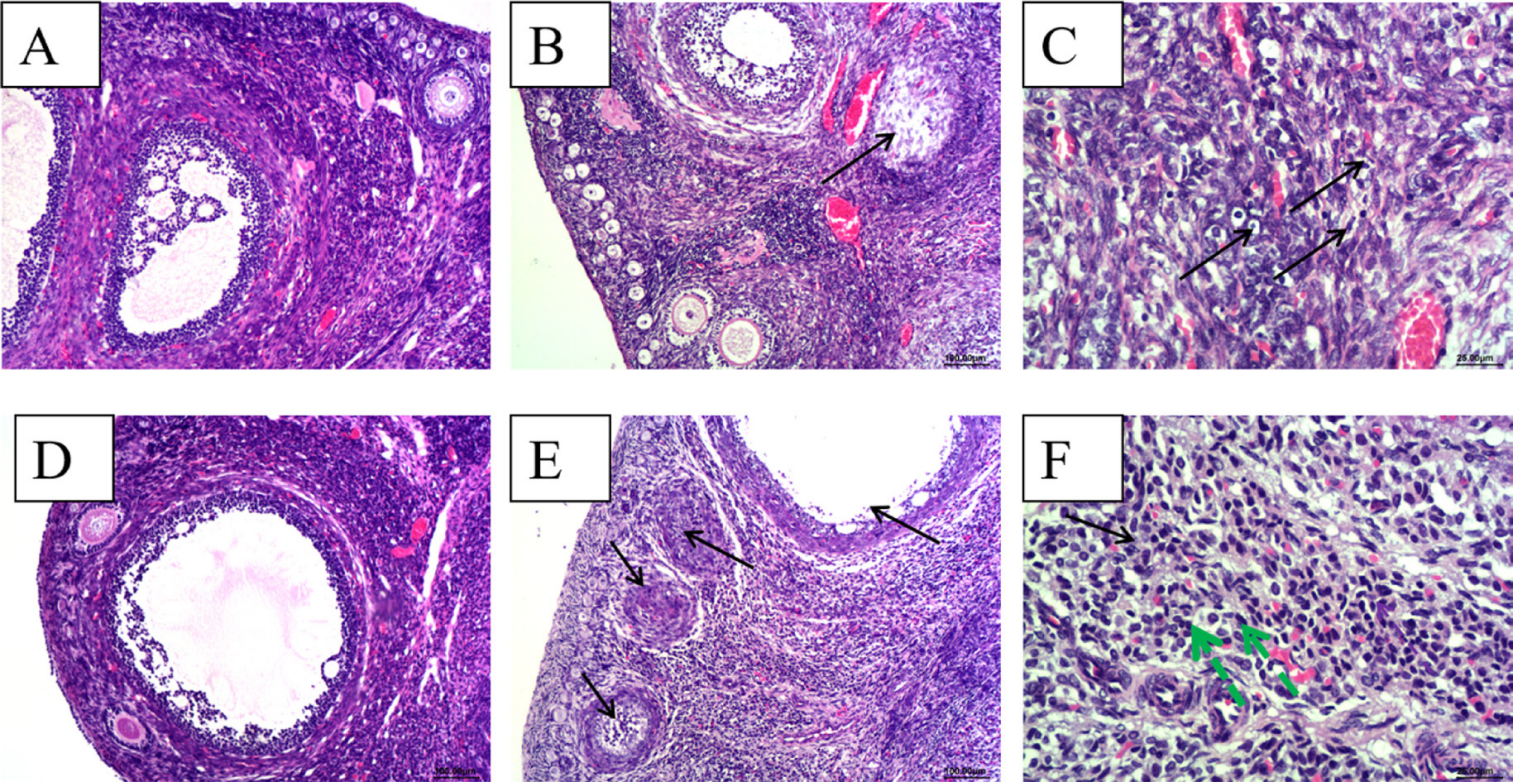
Histopathological analyze of ovaries (**A**) and (**D**), There were no gross histopathological lesions in the ovarian section from the control group at 28 dpi and 49 dpi, respectively.(Magnification: 10×); (**B**) ovarian interstitial cell scattered necrosis (black arrow), a small amount of lymphocytic infiltration in part area of ovarian interstitial from HEV RNA positive rabbit at 28 dpi.(Magnification:10×); (**C**) Necrosis and scattered lymphocytic infiltration (black arrow) in part area of ovarian interstitial from HEV RNA positive rabbit at 28 dpi.(Magnification:40×); (**D**) high power field of the control group (Magnification:20×); (**E**) Large amounts of ovarian germ cell were necrosis (black arrow) and dropped off, an increase of ovarian atresia and lymphpcytic infiltration in the ovarian interstitial from the HEV RNA positive rabbit at 48 dpi. (Magnification:10×); (**F**) Large amounts of lymphcytic infiltration (green arrow) in the ovarian interstitial from the HEV RNA positive rabbit. (Magnification:40×).

Ovarian tissues from control group rabbits were histologically normal, characterized by epithelial cells arranged in order; all levels of the folliculus and ovarian ovum structures were clear and complete (Figure 2A). In contrast, at 28 dpi, for the HEV RNA-positive ovary, epithelial cells in the ovary showed scattered necrosis and dropped off. The follicular cells of primordial follicles showed scattered necrosis (Figure 2B). The ovarian interstitium showed slightly edema and scattered lymphocytic cell infiltration (Figure 2C). At 49 dpi, for HEV RNA-positive ovaries, epithelial cells in the ovary showed high necrosis and dropped off. Large numbers of primordial follicles exhibited necrosis (Figure 2E). Granulosa cells and follicular cells of primary follicles and secondary follicles exhibited obvious necrosis (Figure 2E). The interstitial cells of the ovary showed massive necrosis, and there was a high level of lymphocytic cell infiltration in the stroma of the ovary (Figure 2F).

### Follicular development and expression of estrogen receptor in ovaries

**Figure 3:**
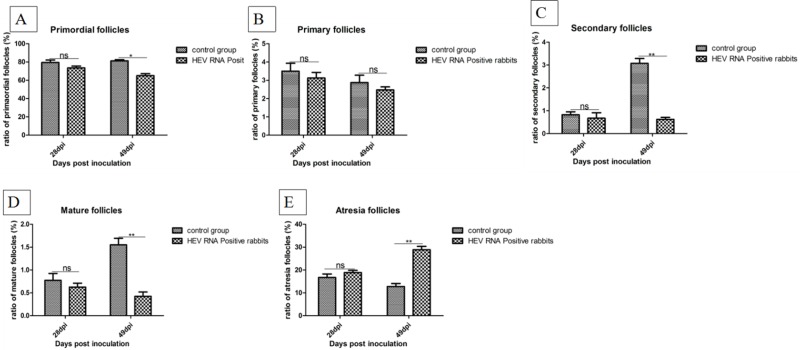
Ratio of follicles at all levels in ovaries (**A**) The ratio of primordial follicles in the ovaries. (**B**) The ratio of primary follicles in the ovaries. (**C**) The ratio of secondary follicles in the ovaries. (**D**) The ratio of mature follicles in the ovaries. (**E**) The ratio of atresia follicles in the ovaries.

The ratios of follicles at all levels in ovaries were counted and the results are shown in Figure 3. Compared to the control group, the ratio of primordial follicles, primary follicles, secondary follicles, and mature follicles was decreased in HEV RNA-positive ovaries, while the ratio of atresia follicles was increased. Expression of the estrogen receptor (ER) was examined by immunohistochemical staining. The ER was mainly distributed in the cytoplasm of interstitial cells of the ovary. The expression levels of ER were measured as the area density of positive substance (Supplementary Figure 4A–4C). The area density of the ER was significantly (*P* < 0.01) higher in HEV RNA-positive ovary tissue than in the control group (Supplementary Figure 5).

### TUNEL assays

**Figure 4:**
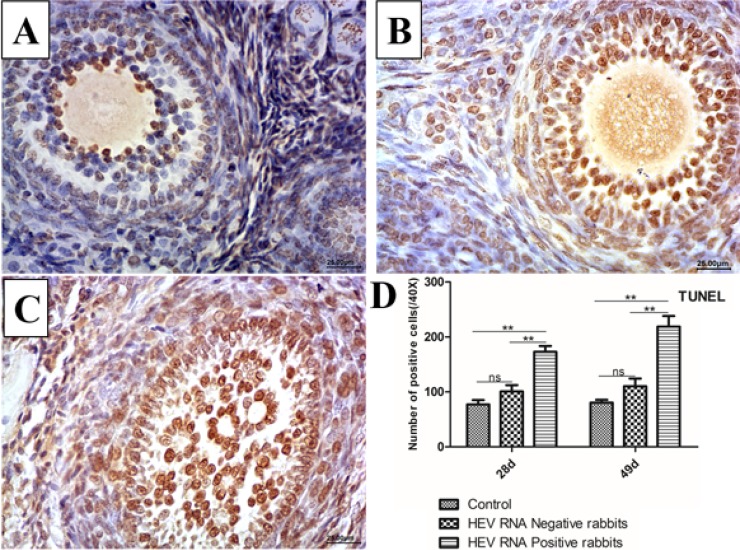
Representative TUNEL-stained histological sections of ovaries from control group and Swine HEV inoculated rabbits TUNEL-positive cells have brown nuclei. The number of TUNEL-positive cells were remarkably higher in HEV RNA positive ovaries at 28dpi (**B**) and 49dpi (**C**) than in the control (**A**). (magnification:40×). Quantitative analysis of TUNEL-positive cells in ovaries of rabbits (**D**). The data were expressed as the percentage (mean ± SD). (^*^P < 0.05) or (^**^P < 0.01), indicated statistical significance versus the control group (*n* = 5).

The TUNEL assay detects DNA fragmentation, a hallmark of apoptosis. Positive signals were observed in the nucleus of apoptotic cells. Compared to the control group (Figure 4A), the TUNEL assay results showed strong positive signals in follicular cells and oocytes at 28 dpi (Figure 4B) and 49 dpi (Figure 4C). In addition, the numbers of apoptotic cells were significantly higher in HEV RNA-positive ovary tissues than in the control group (Figure 4D).

### Expression of Bcl-2, Bax, and Caspase-3 in ovary tissues

**Figure 5:**
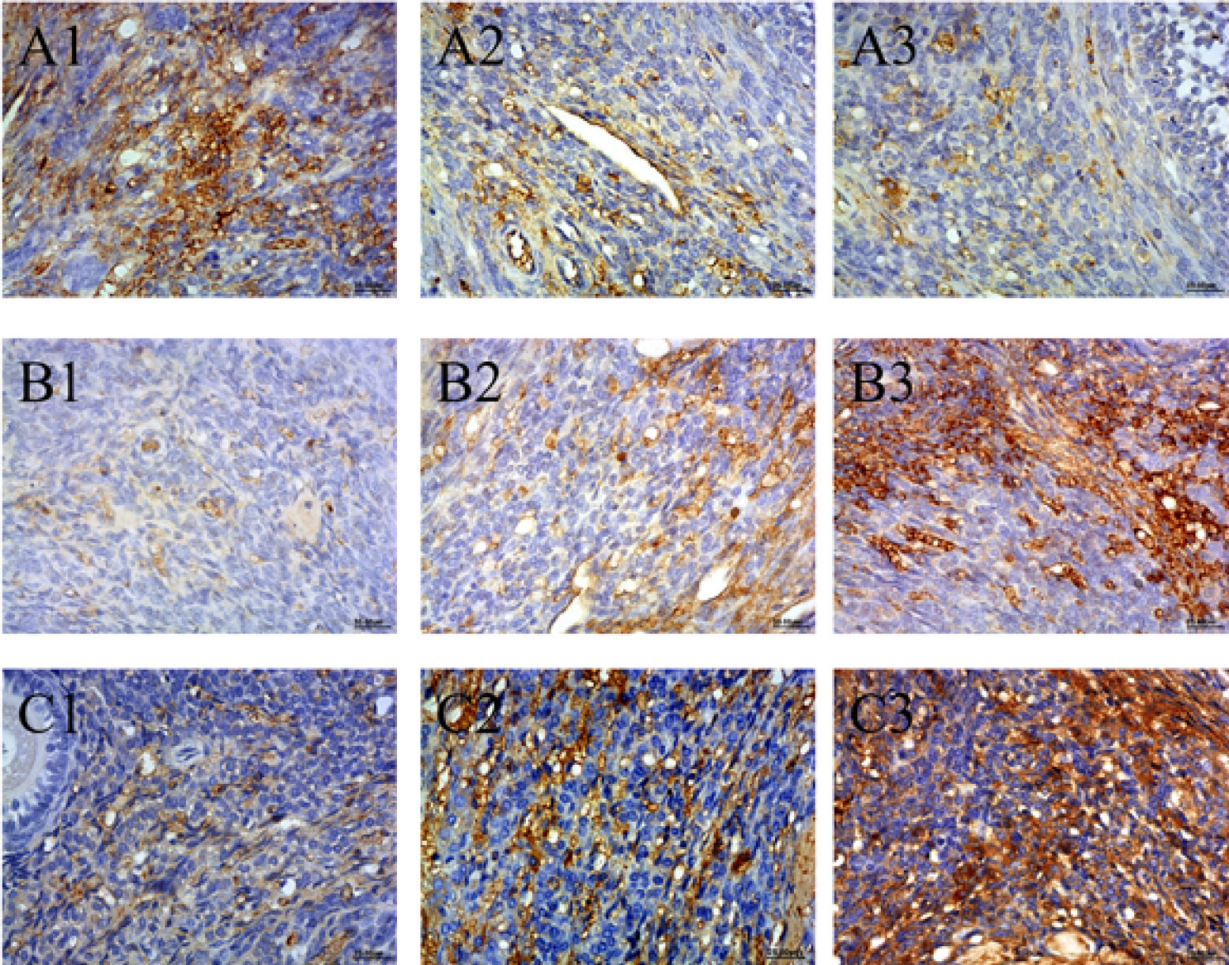
(**A**) Expression of Bcl-2 (A1–A3), (**B**) Bax (B1–B3), (**C**) Caspase-3 (C1–C3) in ovaries of HEV inoculation rabbits. A1, B1 and C1 are control group. A2, B2, C2 are HEV RNA positive ovaries in 28 dpi. A3, B3, C3 are HEV RNA positive ovaries in 49 dpi. (Original magnification:40×).

**Figure 6:**
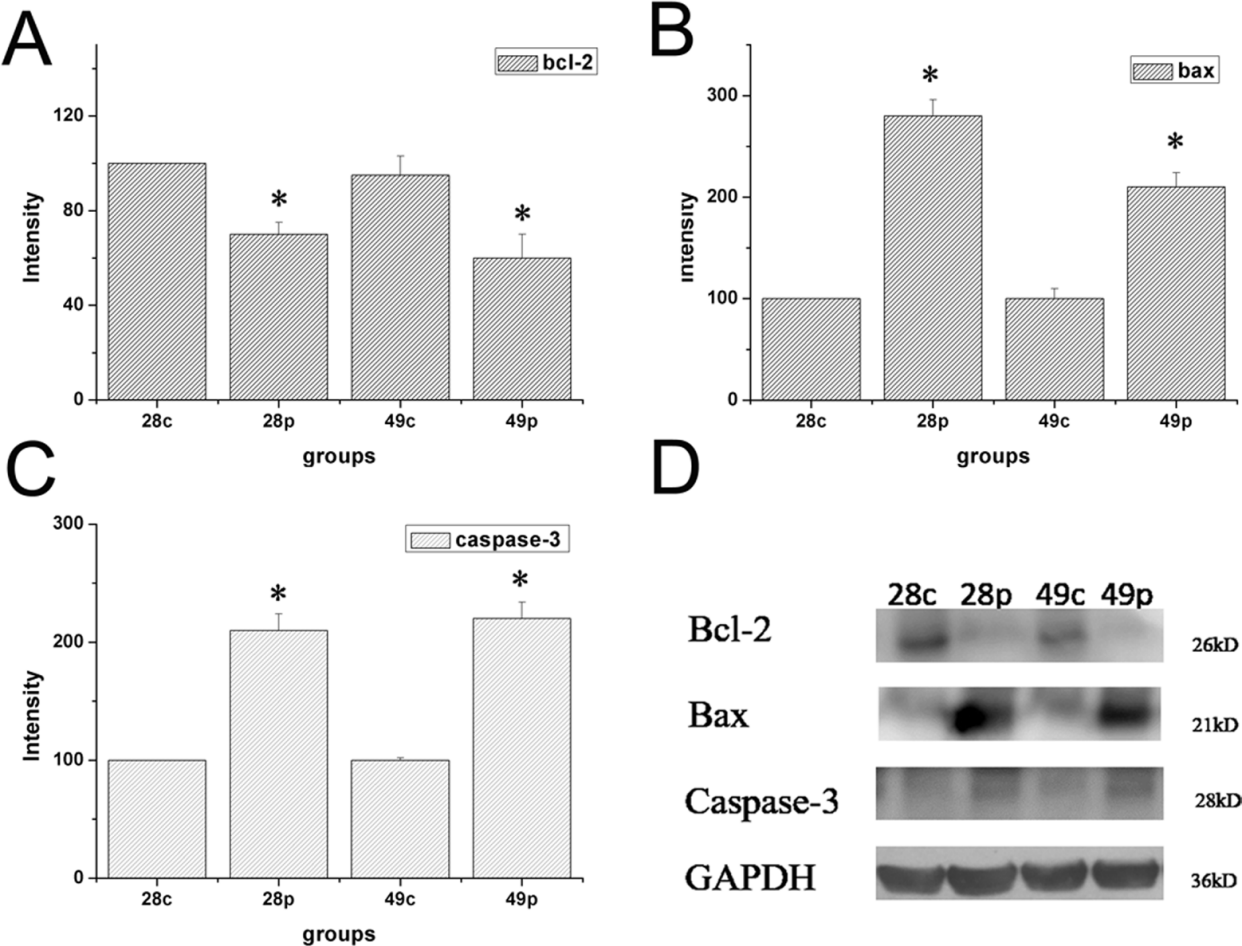
Expression level of protein of Bcl-2, Bax and Caspase-3 in ovary tissue of different groups at 28 dpi and 49 dpi (**A**) intensity of expression of Bcl-2 (**B**) Bax, (**C**) Caspase-3 in ovaries of HEV inoculation rabbits. (**D**) Western blot analysis of Bcl-2, Bax and Caspase-3 in ovaries in different groups.

Expression of Bcl-2, Bax, and Caspase-3 were examined by immunohistochemical staining. The distribution of Bcl-2, Bax, and Caspase-3 was mainly in the cytoplasm of interstitial cells of the ovary. The expression levels of Bcl-2, Bax, and Caspase-3 were measured as the area density of positive substance (Figure 5A–5C). The area density of Bax/Bcl-2 and Caspase-3 was significantly (*P* < 0.01) higher in HEV RNA-positive ovary tissue than in the control group (Supplementary Figure 6). The protein expression levels of Bcl-2, Bax and Caspase-3 were measured by wertern blotting. As shown in Figure 6, compared to the control group, Bcl-2 expression results showed significant decrease of HEV-RNA positive ovary tissue at 28 dpi and 49 dpi (Figure 6A–6D). The expression of Bax and Caspase-3 were significantly higher in HEV RNA-positive ovary tissues than in the control group (Figure 6B–6D).

## DISCUSSION

Although the primary replication site of HEV is the liver, its replication and expression in many extrahepatic tissues has also been reported such as in the gut, kidney, and brain [8, 10, 13, 14]. HEV can replicate in many extrahepatic cells such as neuronal-derived cells, explaining the neurologic disorders observed during HEV infection [17]. The expression of HEV in many other extrahepatic tissues suggests that these cells allow HEV to replicate and are targets of the virus, producing extrahepatic manifestations [7]. As a zoonotic virus, HEV infection has a high mortality rate in pregnant women and vertical transmission is an important route of HEV transmission [18–20]. Previous studies demonstrated that HEV can replicate in the human placenta and gerbil testis [9, 13].

However, as a major tissue of the mammalian reproductive system, the ovary has never been reported as an extrahepatic replication site of HEV. In this study, reverse transcription nested PCR and real-time PCR show that HEV RNA can exist in the ovaries. Because HEV is a positive-strand RNA virus, its replication produces a negative-strand RNA. The detection of negative-strand HEV RNA revealed replication of HEV in the ovaries. Thus, the ovary is a replication site for HEV.

We also detected the ORF2 and ORF3 antigens of HEV by immunohistochemical staining. The results showed positive signals not only in granular cells of the ovary, but also in the ovum at different stages. Previous studies showed that HBV is expressed and distributed in the ovary and ovum, indicating a new mechanism of HBV vertical transmission [21]. Similar to HBV, our results suggest that the HEV antigen present in the existed in the ovum can enter the zygote as soon as the ovum is inseminated, followed by HEV transmission to the embryo. These findings indicate a new mechanism of HEV vertical transmission.

The rate of miscarriage in pregnant women with hepatitis E is approximately 8–16% [22, 23]. Studies of SPF rabbits with HEV infection also showed that HEV is associated with a high incidence of infertility and miscarriage, but the mechanism remained unclear. Based on the results of the present study, the expression of HEV may injure the ovary. Expression of HEV in the ovum may decrease the vitality of the ovum and cause pathological damage to the ovary tissue. Thus, these findings may be helpful for studying the replication mechanism, transmission, and pathogenesis of HEV in the reproductive system and its subsequent effects on fertility. The mechanism of infertility caused by HEV has not been examined previously, and further studies are necessary.

Infertility and miscarriage caused by HEV may be attributed to injury of the ovary tissue. In this study, we also detected the detrimental effects of swine HEV on the rabbit ovaries and observed changes in ovary tissues attributed to virus inoculation. According to histopathological observation, ovarian germ cells exhibit necrosis and drop off, and all levels of follicles show varying degrees of necrosis in HEV RNA-positive tissue. Inflammatory cell infiltration was also observed in the interstitium of the ovary. Histopathological changes in the ovaries tissues following inoculation revealed the presence of HEV in the ovaries. Additionally, the significantly high expression of ER in HEV RNA-positive ovaries demonstrated that HEV infection can cause disorders in hormone secretion. Compared to the control group, the ratios of primordial follicles, primary follicles, and secondary follicles were significantly decreased, whereas the ratio of atretic follicles was significantly increased in HEV RNA-positive ovaries. Ovaries play an important role in mammalian reproduction and development. Severe pathological changes in ovary tissue can induce damage to the ovarian structure. The significant changes in the follicles at all levels and increase in ER demonstrate that HEV infection can damage ovarian function. This may explain the high infertility and miscarriage rates of women with HEV infection.

Previous studies demonstrated that HEV infection can damage the ovary structure, but the mechanism was unclear. Apoptosis is a mode of programmed cell death and is essential for removing cells during developmental processes, including during oocyte development and maturation. Apoptosis of germ cells in ovaries is a normal physiological process and essential for follicle growth. During infection by some pathogens, cell apoptosis increases [24]. Previous studies demonstrated that HEV infection also increases apoptosis both in the kidney and testis. In this study, we observed a significantly higher number of TUNEL-positive cells and higher area density of Caspase-3 and BAX/BCL-2 in HEV RNA tissues compared to in the control group, indicating increased ovarian cells apoptosis. The significantly high number of TUNEL-positive cells observed in infected cells further confirmed the apoptosis of ovarian cells. Under normal conditions, apoptosis also plays a major role in eliminating germ cells at all stages of oogenesis and even after ovulation [25]. In this study, we used rabbits of the same age; at different time points, we selected rabbits from the control group and experimental group to avoid individual differences. The results showed that the ratio of apoptotic cells in the experimental group was generally higher than that in the control group. Therefore, the high apoptotic ratio in the experimental group was not caused by individual differences in this study. Moreover, the high apoptosis rate of infected cells further confirmed that HEV infection and replication in ovarian tissues caused structure damage to cells.

BCL-2 family members play an important role in regulating the mitochondrial pathway of apoptosis and includes inducers and inhibitors of the cell death process [26]. Changes in the BAX/BCL-2 ratio are related to cytochrome C activation, and cytochrome C is released from the mitochondria. Next, cytochrome C triggers caspase activation and apoptosis occurs. In this study, the high expression of BAX and low expression of BCL-2 in infected cells indicated that HEV infection activates mitochondrial pathways and finally induces cell death. However, the pathway of apoptosis caused by HEV and involvement of pro- and anti-apoptotic mechanisms remains unclear. Thus, further studies are needed to elucidate the mechanism of molecular changes associated with pathological injury and accelerated apoptosis caused by HEV infection.

In conclusion, the present study demonstrated that the swine HEV RNA and antigen can exist and replicate in the ovaries of rabbits infected experimentally with HEV. The expression of HEV antigen in the ovum suggests a new mechanism of HEV vertical transmission. Additionally, histological injury and molecular changes such as ovarian interstitial and follicle cell scattered necrosis, lymphocyte infiltration, and accelerated apoptosis in ovarian tissues of rabbits infected with HEV were observed in this study. HEV infection has many different extrahepatic manifestations and further studies are needed to explore the HEV extrahepatic replication mechanism in the ovary. The mechanism of HEV vertical transmission also requires further analysis.

## MATERIALS AND METHODS

### Animals and ethics statement

Sixteen 80-day-old female New Zealand white rabbits in estrus weighing between 1.8 and 2.0 kg were purchased from Xing Long Experimental Animal Center (Beijing, China). Before inoculation, the blood and feces of all rabbits used in the experiment were confirmed to be negative for HEV RNA by reverse transcription-nested PCR (RT-nPCR). The serum was also confirmed to be negative for HEV antigen and HEV antibody in an enzyme-linked immunosorbent assay (ELISA). The use of animals was approved by the China Agriculture University Institutional Animal Care and Use Committee with protocol number 20140115-089.

### Virus preparation

As described in our previous study [8], strain HB-L3 of genotype 4 swine HEV (GenBank No.KJ123761,KX531115), with 90.9% homology to a Beijing human strain (GenBank No. AJ272108), was first used to intraperitoneally inoculate two rabbits at 10 mL per day for 7 consecutive days. When the fecal samples of both rabbits were positive for HEV RNA at 4 weeks post-inoculation, both rabbits continued feeding for one week and then were sacrificed. Samples of liver and intestinal contents were positive for HEV RNA and stored at –80°C. Next, a 10% suspension of intestinal contents was prepared and used as the infectious viral stock. The titer of viral suspension was 6.63 × 10^7^ genome equivalents per mL according to real-time PCR detection.

### Experimental design

Sixteen rabbits were numbered and randomly divided into two groups with 8 rabbits in the infectious group and 8 in the control group. Each rabbit in the experimental group was inoculated intraperitoneally with 10 mL of viral suspension per day for 7 consecutive days. Each rabbit in the control group was injected with an equal volume of negative fecal suspension. Each rabbit was housed in a separate cage and the conditions of all rabbits were monitored every day. No rabbit died following viral inoculation and no clinical symptoms were observed in HEV-infected rabbits.

### Sampling

Four rabbits from each group were sacrificed at 28 and 49 days post-inoculation (dpi). Serum was collected to evaluate the levels of alanine aminotransferase and aspartate aminotransferase. Ovary samples were collected, washed with normal saline, and divided into two parts. One part was fixed in neutral 4% paraformaldehyde solution for histopathological and immunohistochemical analysis, whereas the other part was directly frozen in liquid nitrogen and stored at −86°C until PCR analysis.

### ELISA for HEV antigen detection

Serum samples of rabbits were collected and tested for HEV antigen and anti-HEV IgG using an HEV ELISA kit following the manufacturer's instructions (Wantai, Beijing, China).

### PCR assays

Total RNA was extracted from ovary tissues using the UltraPure^TM^ RNA Kit (CWBIO, Beijing, China). Next, the RNA was reverse-transcribed using the HiFi script cDNA Synthesize Kit (CWBIO) according to the manufacturer's instructions. Primers targeting the HEV ORF2 region consisted of out primers P1, P2 and inner primers P3, P4 and were used as previously described. The PCR parameters included initial denaturation at 95°C for 1 min, 42°C for 1 min, and 72°C for 2 min, with a final extension step at 72°C for 10 min. A 348-base pair PCR product was expected after the second round. Positive ovary tissues were used for real-time PCR to detect the viral load; the detailed protocol has been described previously [17].

Ovary tissues with positive HEV RNA were assayed for negative-sense HEV RNA. Reverse transcription targeting the negative-strand HEV RNA was performed without primer P1. Parental RNA was degraded using RNaseA, followed by nested PCR with the same PCR parameters. Sterile ddH_2_O was included as a negative control.

### Histopathology examination

All fixed ovaries tissues were processed and embedded in paraffin wax, sectioned at 4 μm thickness, and stained with hematoxylin and eosin for histological observation. Ovary morphology was evaluated by light microscopy (Olympus, Tokyo, Japan). The ratios of the number of follicles at all levels in the control group and experimental group were counted (the number of follicles in the whole section was counted).

### Immunohistochemical (IHC) staining

The 4-μm-thick ovaries tissues were subjected to immunohistochemistry staining using 3,3′-diaminobenzidine tetrahydrochloride (ZSGB-BIO, Beijing, China) to visualize the antigen-antibody reaction. Gill's hematoxylin was used as the background stain. The detailed protocol was conducted according to the instructions of the Histostain^TM^-Plus Kit (ZSGB-BIO). Monoclonal mouse anti-HEV ORF2 and ORF3 antibodies (1:200 dilutions; Beijing Protein Innovation, Beijing, China) were used to detect HEV antigen and replication. The expression of apoptotic proteins (BAX, Bcl-2, and Caspase-3; 1:200 dilution; Boster Co., Ltd., Beijing, China) in the ovary were detected by immunohistochemical staining. The expression of ER (1:200 dilution; Boster Co., Ltd.) in the ovary was detected by immunohistochemical staining. The slides were observed under a light microscope (Olympus) and measured using the Motic Med 6.0 CMIAS Image Analysis System (Motic China Group Co., Ltd), as described by Majid Hussain Soomro [14]. Positive signals were observed as a brown or yellow granular mass. The positive staining intensity of BCL-2, BAX, and Caspase-3 protein were measured as the ratio of the stained area to the total field assessed. Multiple views (three fields per section, five sections per rabbit) were randomly selected and analyzed.

### Western blot

We determined the protein expression levels of Bcl-2, Bax and Caspase-3 by Western blot analysis. Densitometric analysis of the Western blots was performed using Image Lab™ software (Quantity ONE7.0). TheBcl-2 and Caspase-3 antibodies were obtained from Biosynthesis biotechnolgoy Co., Ltd.,(China). Bax antibody was purchased from Proteintech Group, Inc (USA).

### TUNEL assay

Apoptotic cells in the ovary tissues were detected by the TUNEL method using a cell apoptosis detection kit (*In Situ* Death Detection Kit, POD Cat. No. 11 684 817 910, Roche Applied Science, 68298 Mannheim, Germany). Apoptotic cells have a brown nucleus, while normal cells a have blue nucleus. Multiple views (five per section of each organ) were used to count the number of TUNEL-positive cells per view.

### Statistical analysis

The data were analyzed for statistical significance using SPSS 18.0 (SPSS, Inc., Chicago, IL, USA) as the means and standard deviation. Differences were considered statistically significant with *P* < 0.05 indicated using one asterisk and *P* < 0.01 using double asterisks. All graphs were generated with GraphPad Prism 5.0 (GraphPad Software, La Jolla, CA, USA).

## SUPPLEMENTARY MATERIALS FIGURES


