# Supplementary material for: Replication of hepatitis E virus in the ovary and promotion of oocyte apoptosis in rabbits infected with HEV-4
Source: Oncotarget. 2017 Dec 17;9(4):4475–84. doi: 10.18632/oncotarget.23381 (PMC5796988; doi:10.18632/oncotarget.23381)
Supplement: Supplementary file 1 [file oncotarget-09-4475-s001.pdf]

Replication of hepatitis E virus in the ovary and promotion of oocyte apoptosis in rabbits infected with HEV-4

SUPPLEMENTARY MATERIALS

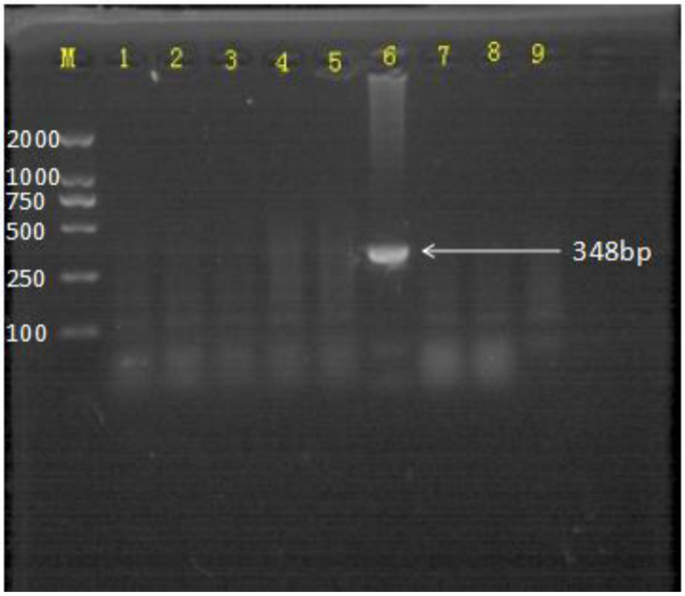

Supplmentary Figure 1: RT-nPCR assays for positive-strand HEV RNA in ovaries at 28 dpi; 1–4:Control group rabbits' ovaries; 5–8: Experimetntal group rabbits' ovaries; 9:negative control; M, marker (From 100 bp to 2000 bp)

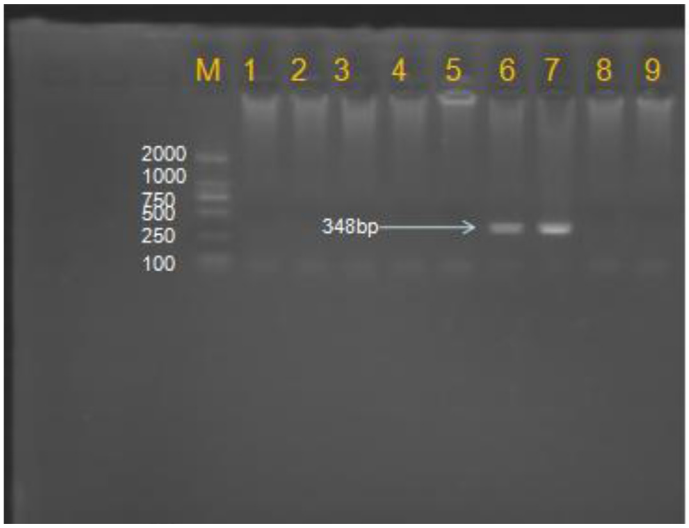

Supplmentary Figure 2: RT-nPCR assays for positive-strand HEV RNA in ovaries at 49 dpi; 1–4:Control group rabbits' ovaries; 5–8: Experimetntal group rabbits' ovaries; 9:negative control; M, marker (From 100 bp to 2000 bp).

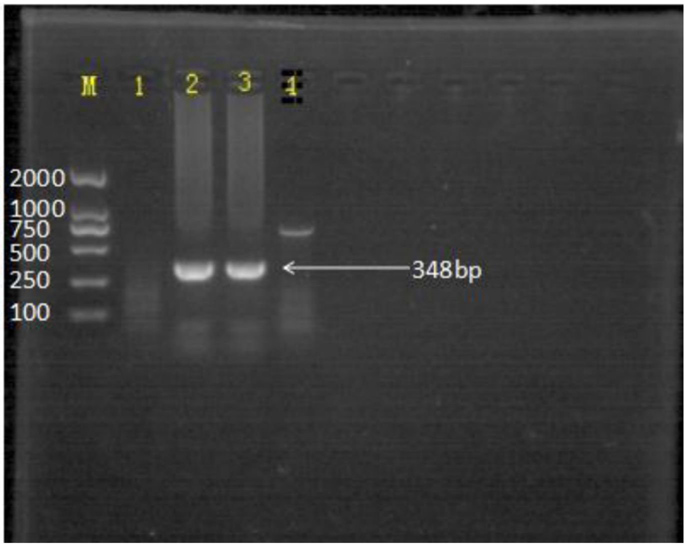

Supplmentary Figure 3: RT-nPCR for negative-strand HEV RNA in ovaries at 28 dpi and 49 dpi. 1:Positive-strand HEV RNA positive sample at 28 dpi; 2-3:Positive-strand HEV RNA positive samples at 49 dpi; 4: negative control; M, marker (from 100 bp to 2000 bp).

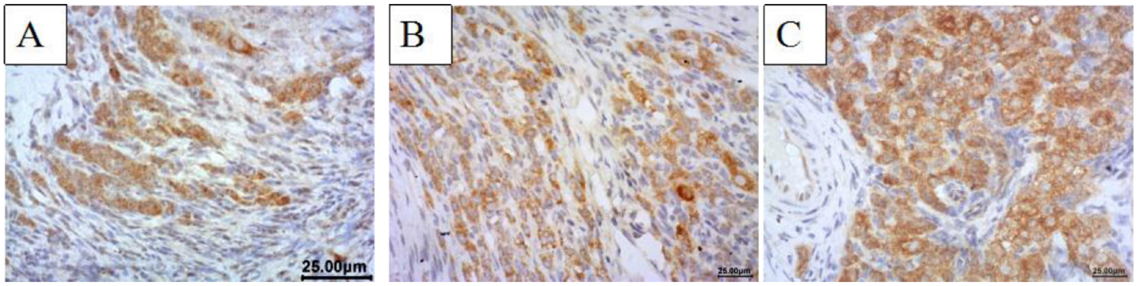

Supplmentary Figure 4: Immunohistochemical stain of ER antigen. (**A**) Expression of ER antigen in control group. (**B**) Expression of ER antigen in HEV RNA positive rabbits at 28 dpi. (**C**) Expression of ER antigen in HEV RNA positive rabbits at 49 dpi. (Magnification: 40×).

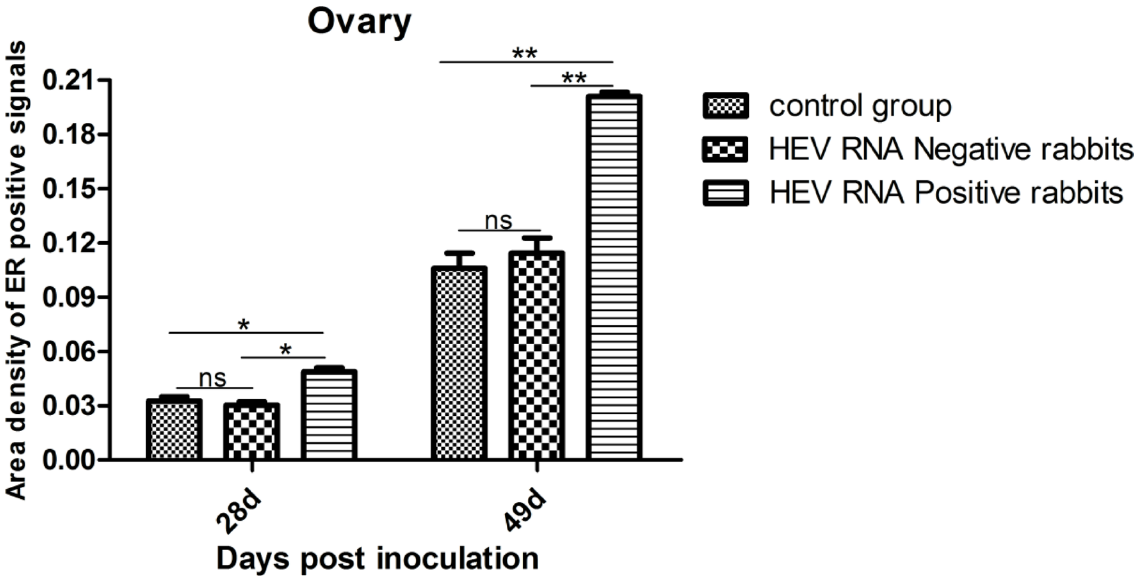

Supplmentary Figure 5: Area density analysis of ER expression in ovaries is indicated in above figure. Data are shown as mean ± SD. $^{*}P$ significant (P values < 0.05) and $^{**}P$ significant (P values < 0.01).

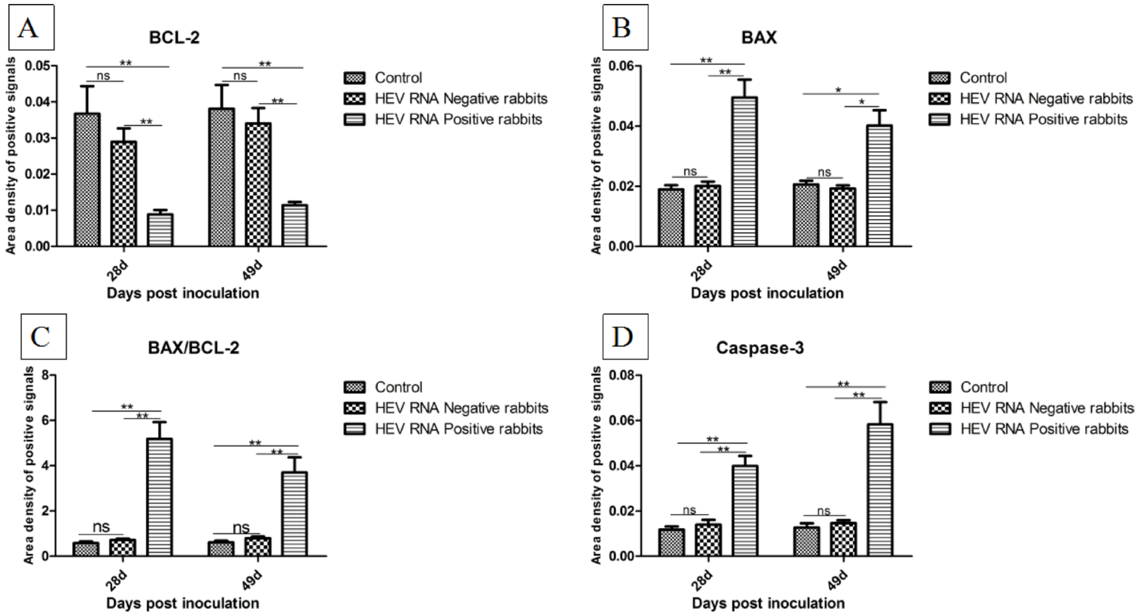

Supplmentary Figure 6: Area density analysis of apoptotic protein expression levels of BCL-2 (**A**), BAX (**B**), BAX/BCL-2 (**C**), caspase-3 (**D**) in ovaries is indicated in above figure. Data are shown as mean ± SD. $^{*}P$ significant (P values < 0.05) and $^{**}P$ significant (P values < 0.01).
